# Supplementary material for: The Impact of the COVID-19 Pandemic and Associated Societal Restrictions on People Experiencing Homelessness (PEH): A Qualitative Interview Study with PEH and Service Providers in the UK
Source: Int J Environ Res Public Health. 2022 Nov 23;19(23):15526. doi: 10.3390/ijerph192315526 (PMC9739517; doi:10.3390/ijerph192315526)
Supplement: Supplementary file 1 [file ijerph-19-15526-s001.zip › ijerph-1982241-supplementary.pdf]

Interview topic guide: Homelessness Group

Ask to describe 'normal life' before the pandemic

- Living situation
    - Where were you living?
- How long have you been homeless for?
- Employed? Type of job, hours etc
- Education/study?
- Parent or carer?
- Use of any community services?
- If they have any health conditions they mentioned (what condition, when diagnosed, if on treatment or whether they have had complete treatment)
    - Any substance use – alcohol/drugs? Any support for these if so?
    - What was treatment plan? How was it being managed? What was usual routine for appointments/follow-up?
- Are you currently registered with any support services? (GP, third sector etc).

UNDERSTANDING AND ADHERENCE TO GUIDELINES

At the moment, are you self-isolating (how long for, reasons for this), social distancing/ 'staying at home'?

- Please describe what this involves for you?
    - i.e. are you self-isolating with others helping to get essentials, or going out for these?
    - Or self-isolating with no outside exercise? Etc – if self-isolating without outside exercise how are you feeling about this? Do you have access to a garden or outdoor space?

- **What do you understand by the 'social distancing' advice that is being given – what does it mean to you?** Have you been…
    - Avoiding crowds
    - Keeping personal distance from others
    - Isolating
    - Avoiding close contact greetings
    - Socialising/going out only with those in your household
    - <if exercising outside> are you finding places to go where you can keep your distance from others?

Have you been able to stick to the social distancing advice that has been given to your group? Please tell us about why/ why not?
[COM-B prompts can be used here, to include:]

- How has your experience of homelessness impacted your ability to follow social distancing guidelines?
    - Interactions with public?
    - Living situation?
- Any existing physical or mental health problems
- Group membership
- Beliefs about consequences/ health beliefs
- Consequences for others/ self
- Needing to work/ living arrangements, whether others are self-distancing in the same house/area
- Work/ Caring responsibilities, providing emotional support
- Peer pressure to socialise

- Government rules/punishments <prompt to ask how they feel about the Government recommendations>,
- Feelings about losing normal life
- Change of routine/ habits
- Any impact on your ability to access support?

LIVING SITUATION

- Has your living situation changed during the pandemic and if so how?
- Have you received any support with housing during the pandemic? (e.g. charitable/community resources (e.g night shelters) hotels, temporary/emergency accommodation/third sector support)
- Have you felt comfortable in your accommodation during the pandemic? (e.g. Overcrowded/unsafe/unhygienic?
- Has your accommodation had sufficient facilities during the pandemic, including sanitation (showers, sinks etc) and cooking facilities?

ACCESS TO DAILY PROVISIONS/SANITATION

- How has the pandemic affected your ability to access sanitation facilities? (e.g. closure of public toilets/ restaurant facilities, wash and shower facilities etc)
- How has the pandemic affected access to food (including cooking facilities)? Do you feel secure in your ability to access food?
- Have you been able to access support from charitable/community resources during the pandemic? (e.g. soup kitchen/drop in/food banks) Are these different to the resources you would have accessed before?
- Access to PPE, face masks etc

INTERACTION WITH PUBLIC

- Any changes in attitudes or behaviour of others toward you?
- Changes in number of interactions (due to less footfall)?
- Changes in ‘type’ of person interacted with? (e.g. less workers passing through)
- Any changes in money or other donations?
- Any changes in ability to obtain donations/money from public during the pandemic? (if street homeless)
- How do you feel about taking donations off people/interacting with the public/behaviour of the public toward you?

HEALTH CONDITIONS (f applicable)
How has Covid-19 had an impact on the [health condition]?
Prompts include:

- What has been the impact on any normal appointments? (cancelled, delayed, unable to speak to appropriate healthcare professional, changed to different method/location of appointment e.g. online/telephone)
- What has been the impact on any treatment? (cancelled, delayed, changed from usual treatment plan, unable to get medication/prescriptions)
- Have you experienced an impact on any symptoms/side effects?
- How have you felt about [any mentioned changes/impact above]?
- Substance use – any changes in use? Access to services or medication?

SOCIAL LIFE

How would you describe your social life before the Covid-19 pandemic?

- How would you describe your social network – for example size, types of people, types of relationships, do they live with you, nearby or further away, how often do you see each other, how well do you know each other? How do you interact, face to face, online or social media?
- Social activities?
- Could you describe any community services/participation or volunteering participation?
- Could you describe the social/peer support you have? (such as emotional support, advice and information, someone to help you with money or milk/bread/essentials, community services)
- Can you tell us about any ways your social networks/ friendship groups influence you, such as peer pressure, or encouraging you to get involved in things? Do you compare your life to theirs?
- Social engagement (social roles, bonding, attachment)

How would you describe your social life now that social distancing measures have been brought in because of Covid-19? Please tell us about this

Prompts include:

- How would you describe your social network – for example size, types of people, types of relationships, do they live with you, nearby or further away, how often do you see each other, how well do you know each other? How do you interact, face to face, online or social media?
- Social activities?
- Could you describe any community services/participation or volunteering participation?
- Could you describe the social/peer support you have? (such as emotional support, advice and information, someone to help you with money or milk/bread/essentials, getting medication/access to healthcare, community services)
- Can you tell us about any ways your social networks/ friendship groups influence you, such as peer pressure, or encouraging you to get involved in things? Do you compare your life to theirs?
- Social engagement (social roles, bonding, attachment)
- How has your experience of homelessness (**OR** financial hardship/substance use/domestic abuse) impacted your social life/networks during the pandemic?

MENTAL HEALTH

How do you feel about the changes that have been brought about by Covid-19? Have they had any impact on your mental health or wellbeing? Please tell us about these

- What are the things most bothering you at the moment?
- Have you experienced any impact on positive emotions? (prompts: how deeply you can engage with what you are doing, sense of meaning/ purpose, relationships with others, how well you are managing and feelings of control over your situation?)
- Has there been any impact on your sense of identity?
- Have you experienced any negative psychological feelings? (prompts: such as shame, guilt, lack of pleasure, anxiety, worry)
- Please tell us about any physical symptoms due to being stressed or anxious? (prompts: fatigue, sleep problems, pain, illness symptoms, palpitations)
- Has your experience of homelessness (**OR** financial hardship/substance use/domestic abuse) during the pandemic affected your mental health and wellbeing? If so, how?

Have you been doing/ planning anything to help with this?

- Connecting with family or friends?
- Online groups?
- Hobbies/reading
- Exercise <ask about what they have been doing and if there are specific resources they have found useful to exercise>
- Volunteering
- Other engagement

Why are you doing/ not doing these things?

- Helpful/ not helpful – please tell us why
- Enjoyable
- Good for mental health/ wellbeing
- Can't get online, not connected, not comfortable, affordability, confidence in using/ skills
- Skills in using the internet/ communication software
- Living arrangements/ Work/ caring demands
- Peer support/ pressure
- Difficulties/ restriction in physical environment

PROSPECTION
Has the pandemic meant that you have any worries for the future?

How are these different from the worries you had before?

- Sense of control/ powerlessness
- Severity of worries / perspective

Will this change the way you live your life in future?

- The way you connect with others
- How you look after yourself
- How you support others
- How you work?
- How you exercise?

Has this changed any of your priorities for the future?

Is there anything else you would like to tell us about your experiences during the COVID-19 pandemic?
